# Trends in maternal mortality and stillbirths by county in health facility data, Kenya, 2011-2022

**DOI:** 10.1186/s12884-025-07726-6

**Published:** 2025-09-11

**Authors:** Rose Muthee, Martin Mutua, Helen Kiarie, Hannah Kagiri, Edward Serem, Simon Muchemi, Scolastica Wabwire, Ties Boerma

**Affiliations:** 1 Ministry of Health, Nairobi, Kenya; 2 African Population and Health Research Center, Nairobi, Kenya; 3 University of Manitoba, Winnipeg, MB Canada

**Keywords:** Maternal mortality, Stillbirths, Health facility data, Data quality, Kenya

## Abstract

**Background:**

Reports on maternal deaths and stillbirths in health facilities are a critical but underutilized source of information to monitor the quality of care. In addition, with increasing coverage of deliveries by health facilities, such data can improve population estimates of maternal mortality and stillbirth rates. Data quality concerns, however, have often deterred use of facility data. This study aims to assess subnational trends in institutional mortality and examine its utility for improving population-based estimates of mortality.

**Methods:**

Data from the routine monthly reporting system of the Ministry of Health in Kenya were used to assess levels and trends in maternal mortality and stillbirth rates in 47 counties from 2011 to 2022. Data quality was assessed using multiple methods, including consistency of annual reporting of live births, stillbirths and maternal deaths by counties, plausibility of the ratio of reported stillbirths to maternal death, the county institutional mortality in comparison to delivery coverage, socioeconomic development and health system characteristics. The consistency between institutional and population estimates of mortality was assessed using different scenarios.

**Results:**

Institutional live birth coverage increased from 64.0% in 2014 to 87.8% in 2022, ranging from 49 to 99% in counties. Kenya and 39 of its 47 counties experienced a decline in institutional maternal mortality ratio and stillbirth rate during the study period 2011–2022. The national institutional maternal mortality decline stagnated from 2018 and was 99 maternal deaths per 100,000 live births in 2022. Consistency of reported data by county was good over time but several indicators suggest that maternal death reporting was incomplete and more so in less-developed counties. Estimates of the population maternal mortality ratio, derived from the facility data, were much lower than global estimates or census results, while the stillbirth rates were consistent.

**Conclusion:**

The health facility data on maternal death and stillbirths are an important data source for monitoring national and subnational institutional maternal mortality and stillbirth rates and can also inform population estimates. Systematic sustained assessment of reporting completeness will be critical to achieve the full potential of facility data-derived mortality monitoring.

**Supplementary Information:**

The online version contains supplementary material available at 10.1186/s12884-025-07726-6.

## Introduction

Maternal mortality and stillbirth statistics are critical indicators of the risks of childbearing, strength of the health system and country progress in health and development but are difficult to measure accurately. In the absence of complete high-quality death registration systems, most low- and middle-income countries rely on household surveys, and to a lesser extent population censuses, as the main sources of maternal mortality and stillbirth rates in the population. Maternal mortality and stillbirth statistics generated from surveys have major drawbacks, including poor reporting of deaths, low frequency of data collection, high cost of implementing, long reference periods, large confidence intervals and no subnational data for the resulting mortality statistics [1–4]. Censuses are used less frequently and considered a crude instrument for the measurement of maternal mortality [5].

In Kenya, the most recent survey data are from the Demographic and Health Survey (DHS) 2014, where the maternal mortality was 362 per 100,000 live births (95% confidence interval: 254–471) for the seven-year period preceding the survey [6]. The 2022 DHS did not include a maternal mortality module. The 2019 census included a module on recent deaths in the household which resulted in a maternal mortality of 355 per 100,000 live births, with county estimates ranging from 67 in Nyeri to 641 in Garissa [7]. The UN global estimate for Kenya was 530 per 100,000 live births (80% uncertainty interval: 382–750) in 2020, about twice as high as the estimates for neighboring Tanzania and Uganda, and with no decline in the past two decades [8]. The national stillbirth rate was 13 per 1,000 births in the Kenya DHS 2014 and the UN global estimate for 2021 was 18.5 per 1,000 births (90% uncertainty interval: 17.4–19.9) [9]. 

Health facility data on maternal mortality and stillbirths should be used for annual and subnational institutional mortality statistics which are important indicators of the quality of care. Furthermore, if coverage of institutional deliveries is high, the facility data may improve estimates of population levels of maternal mortality. However, the use of health facility data has been limited. Data quality issues, such as completeness of reporting, have been an impediment to the use of facility data for mortality statistics.

The Kenya routine health information system (KHIS) includes monthly aggregate reporting of the number of maternal deaths and stillbirths from all health facilities. Since 2017, individual level data on maternal deaths are also reported by all facilities through the maternal and perinatal death surveillance and response (MPDSR) system. This is a continuous system, reporting facility deaths within 48 h.

This study aims to assess the levels and trends in national and county-level maternal mortality in health facilities during 2011–2022, including an emphasis on data quality, and explore the use of facility statistics in estimates of population mortality.

## Methods

Kenya is administratively divided into 47 counties as part of the devolution in 2013. With a devolved health system, the Ministry of Health deals with policy and research issues while counties are tasked with service delivery. According to the 2019 population census [10], the average population of a county was about 1 million, with Nairobi as an outlier at 4.3 million, as well as eight counties with populations of less than 500,000.

Kenya was one of the first countries in sub-Saharan Africa to deploy an online health information system in 2011. The current KHIS is based on the web-based District Health Information System software (DHIS2). [11] (https://dhis2.org/) Standardized monthly aggregate data reported by health facilities are entered at the county level and include the reporting on numbers of deliveries, live births, stillbirths and maternal deaths. County and national officers verify, analyze and use the data. Data are also reported on neonatal deaths but were not included in this study, as reporting appeared to be poor.

In 2016, the Ministry of Health revised the Maternal and Perinatal Death Surveillance and Response (MPDSR) guidelines to improve the implementation of maternal and perinatal death and “near-miss” reviews at health facilities and in the community. In 2017, the MPDSR started with continuous individual level reporting with notification of maternal deaths within 24 h and perinatal death within 48 h after occurrence through the KHIS.

Annual totals of the monthly numbers of livebirths, stillbirths, and maternal deaths in the KHIS by county were assessed for consistency over time by comparing the index year with an expected value, defined as the median of two preceding and two following years. Imputations were made for extreme outliers with the expected value for that year. For reported numbers of live births, no extreme outliers were identified. For maternal deaths, six annual values (1.1% of the 564 values for all counties during the whole period) differed at least 200% from the expected value which were considered implausible. However, based upon inspection of the time series imputations were made for only two county years with extreme outliers. For stillbirths, imputations were made to 28 county-years with extreme values (> 100% from expected value), almost all (26) occurring during 2011–2014. Details are provided in Appendix A. Our analysis included a comparison of the reported number of maternal deaths by county from the two independent systems of facility reporting, the monthly aggregate KHIS and daily continuous MPDSR, for 2017–2022. We expect the annual totals to be the same and considered higher numbers more complete than lower numbers of reported deaths. Stillbirth (and early neonatal death) reporting is still limited in the MPDSR. Here, we used KHIS data only. Community deaths may also be included in the MPDSR, but such reporting has been limited and is ignored in our analysis.

There is considerable overlap in the causes of stillbirths and maternal deaths [12]. Therefore, we expect the stillbirth to maternal death ratio within a specific range. Based on a review of hospital studies, which reported a range of 7–24 (median 9), we considered ratios between 6 and 30 as plausible (Appendix B). We examined the ratio of the number of institutional stillbirths to maternal death as an indicator of data quality. If the ratio is improbably high, maternal deaths are likely underreported (or less likely stillbirths are overreported). If the ratio is improbably low, stillbirths may be underreported or maternal deaths overreported. A plausible ratio may mean that both events are well-reported, or both are underreported. In all cases, the observed stillbirth rate itself may provide further information on which of the biases is predominant: levels of 15 per 1,000 births or higher were considered as suggestive of no or limited underreporting.

For the assessment of county trends, we synthesized the annual estimates for three four-year periods: 2011–2014, 2015–2018, 2019–2022. For the most recent period, we classified the counties into four groups based on coverage of live births by health facilities in Kenya DHS 2022: [13] < 60%, 60–84%, 85–94% and 95–100%. We examined the associations with county levels of poverty headcount ratio [14] (2021, Kenya National Bureau of Statistics), percent of women 15–49 years with at least secondary education (DHS 2022), county total fertility rates for 2020–2022, DHS 2022), core health professionals (physicians, nurses, midwives) density per 10,000 population (2022, Ministry of Health databases), hospital beds per 10,000 population (2022, Ministry of Health databases).

We estimated the population maternal mortality ratio (MMR) from the institutional MMR (iMMR), coverage of live births by health facilities and assumptions of the community to institutional mortality ratio (M_c_/M_i_ ratio), based on the following association:

$$\:{M}_{p}={P}_{i}*{M}_{i}+\left(1-{P}_{i}\right)*{M}_{c}$$

This implies that

$$\:{M}_{p}=\:Mi*(Pi-\left(1-Pi\right)*\:\frac{Mc}{Mi})\:$$

Where *M*_*P*_ = maternal mortality ratio in the population; *M*_*i*_ = institutional maternal mortality ratio; *M*_*c*_= maternal mortality ratio in the community; *P*_*i*_ the proportion of live births in institutions.

The reference values of M_c_/M_i_ ratios for maternal mortality were derived from a review of studies that included information on place of maternal death and place of delivery (health facilities or not) or mortality statistics for community and health facilities (Appendix C). In this analysis, we conducted a sensitivity analysis using scenarios with ratios of 1, 1.5, 2 and 3. The same computations were done for the stillbirth rate (SBR, iSBR for institutional mortality), using the same mortality ratio scenarios, although empirical data on the ratio are limited.

## Results

### Institutional deliveries

**Fig. 1:**
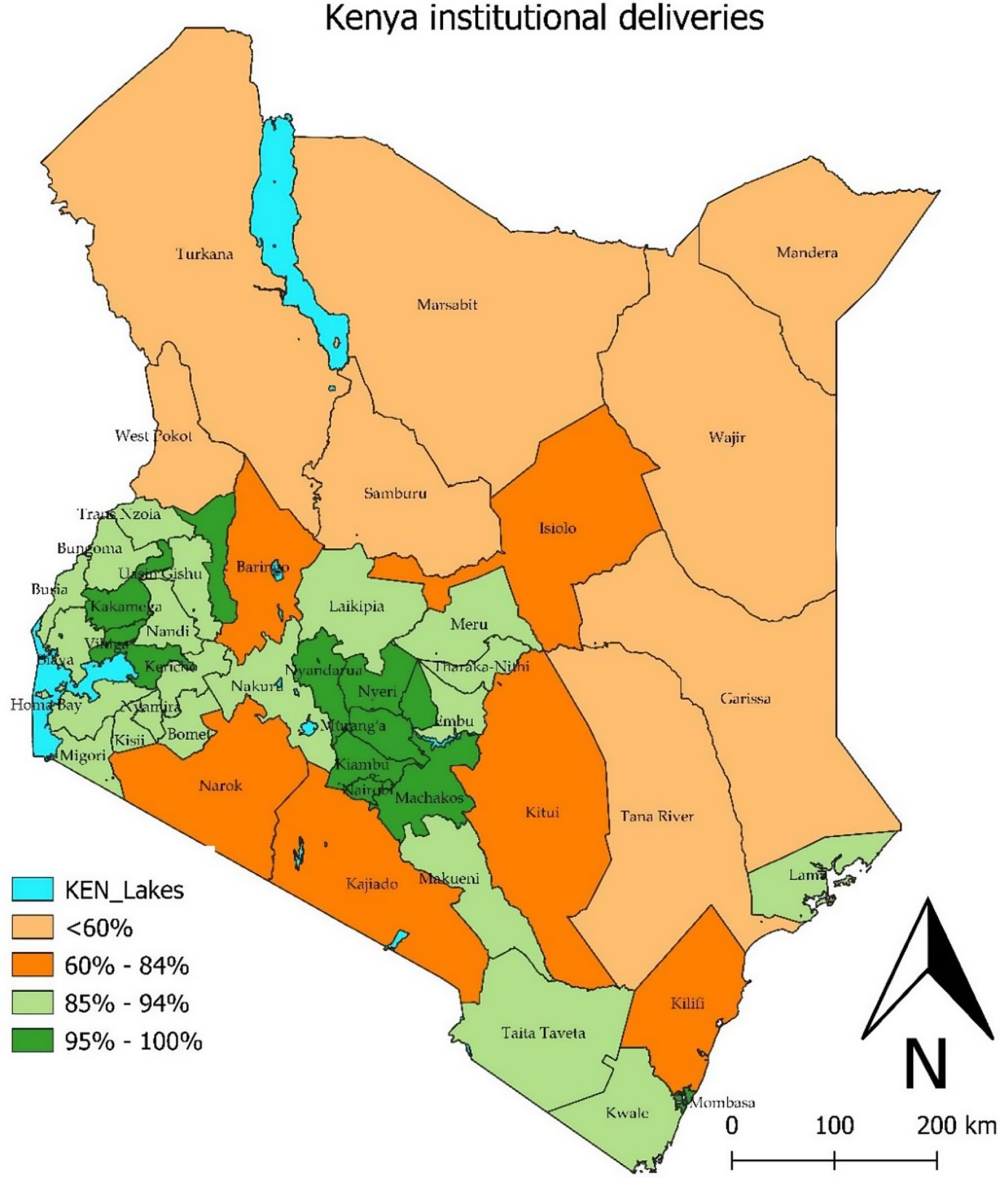
Map of counties with coverage of institutional live births in the two years preceding the survey, DHS 2022, Kenya

Kenya experienced a major increase in coverage of live births in health facilities from 64.0% to 87.8% in the two years preceding the DHS 2014 and DHS 2022, respectively. Coverage increased in all counties (Appendix D). There was considerable variation in coverage between counties in DHS 2022. According to the recent survey, eight counties, all located in the northern and northeastern Kenya, still had coverage of live births below 60%. At the other extreme, near universal coverage (at least 95%) was observed in 11 counties, mostly located in central Kenya (Fig. 1).

Counties in the lowest coverage group had substantially more poverty, lower levels of female education, higher fertility, and poorer health system indicators than those in the higher coverage groups, based on data for 2019–2022 (Table 1, panel A).

**Table 1:** Characteristics of counties (upper panel), and stillbirth rates and maternal mortality ratios (lower panel), grouped by coverage of live births by health facilities, Kenya, 2019–2022

	Coverage of live births by health facilities				
	49–60%	60–84%	85–94%	95–100%	Kenya
Number of counties	8	6	22	11	47
Coverage of births by health facilities (median, DHS 2022)	52	81	92	97	88
*A: Characteristics of the county groups (county medians)*					
Population living below poverty line, 2021 (%)	67	48	36	33	39
Women 15–49 with at least secondary education, 2022 (%)	21	47	55	60	58
Total fertility rate, 2020–2022 (children per woman)	6.2	4.0	3.4	3.0	3.4
Health workforce per 10,000 population, 2022	5.0	7.0	8.3	10.9	8.7
Hospital beds per 10,000 population, 2022	5.0	8.9	9.4	15.5	11.6
*B: Institutional mortality*,* 2019–2022 (medians)*					
Stillbirths per 1,000 births in facilities (range)	24 (15–32)	21 (19–24)	20 (11–27)	18 (16–22)	18
Maternal mortality per 100,000 live births in facilities (range)	111 (72–271)	95 (56–165)	95 (26–118)	92 (34–206)	104
Ratio stillbirths to maternal death in facilities -median (range)	19 (9–38)	22 (14–34)	20 (10–44)	20 (11–58)	18
*C: Scenarios: Population maternal mortality per 100*,*000 live births*,* based on institutional mortality*,* coverage of live births by institutions*,* and assumptions on community to institutional mortality ratio*,* 2019–2022*					
Ratio = 1.0	111	95	94	92	104
Ratio = 1.5	137	108	98	93	110
Ratio = 2.0	164	121	101	94	117
Ratio = 3.0	220	146	109	97	129
*D: Scenarios: Population stillbirths per 1*,*000 live births*,* based on institutional mortality*,* coverage of births by institutions*,* and assumptions on community to institutional mortality ratio*,* 2019–2022*					
Ratio = 1.0	24	21	20	18	19
Ratio = 1.5	30	23	21	18	20
Ratio = 2.0	35	25	22	19	22
Ratio = 3.0	46	29	23	19	23

### National and County trends in institutional mortality

**Fig. 2:**
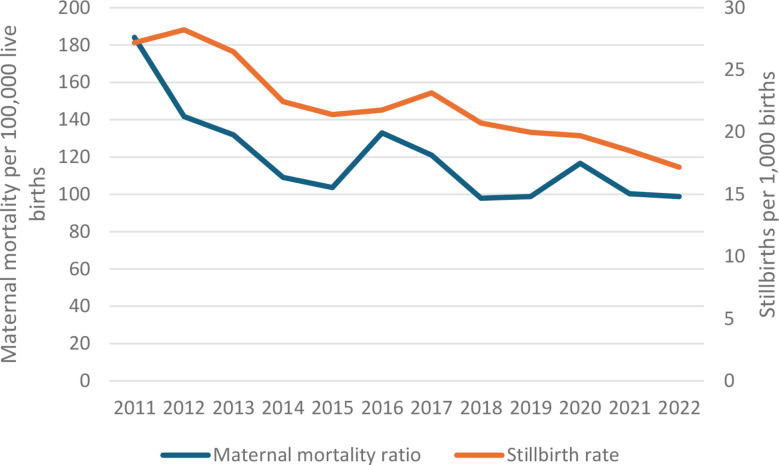
Maternal mortality per 100,000 live births and stillbirths per 1,000 births in health facilities, Kenya routine health information system (KHIS), 2011–2022

During the period 2011–2022, KHIS included 13,075 maternal deaths, 243,995 stillbirths and 11,324,452 live births, after corrections for major outliers. The median annual reported number of maternal deaths varied from 13 to 23 by county, and 26% of county years had less than 10 deaths. The national iMMR was 99 per 100,000 livebirths in 2022, declining during 2011–2018 but little progress since (Fig. 2). A 17% increase was observed in 2020 during the COVID pandemic compared to 2019 and 2021. The iMMR based on the MPDSR reports was 115, 109 and 116 per 100,000 live births for 2020, 2021 and 2022, respectively.

The iSBR reduced from 28 in 2012 to 17 per 1,000 live births in 2022. The national ratio of stillbirths to maternal death declined from 24 in 2011-14, to 20 in 2015-18 and 18 in 2019-22.

**Fig. 3:**
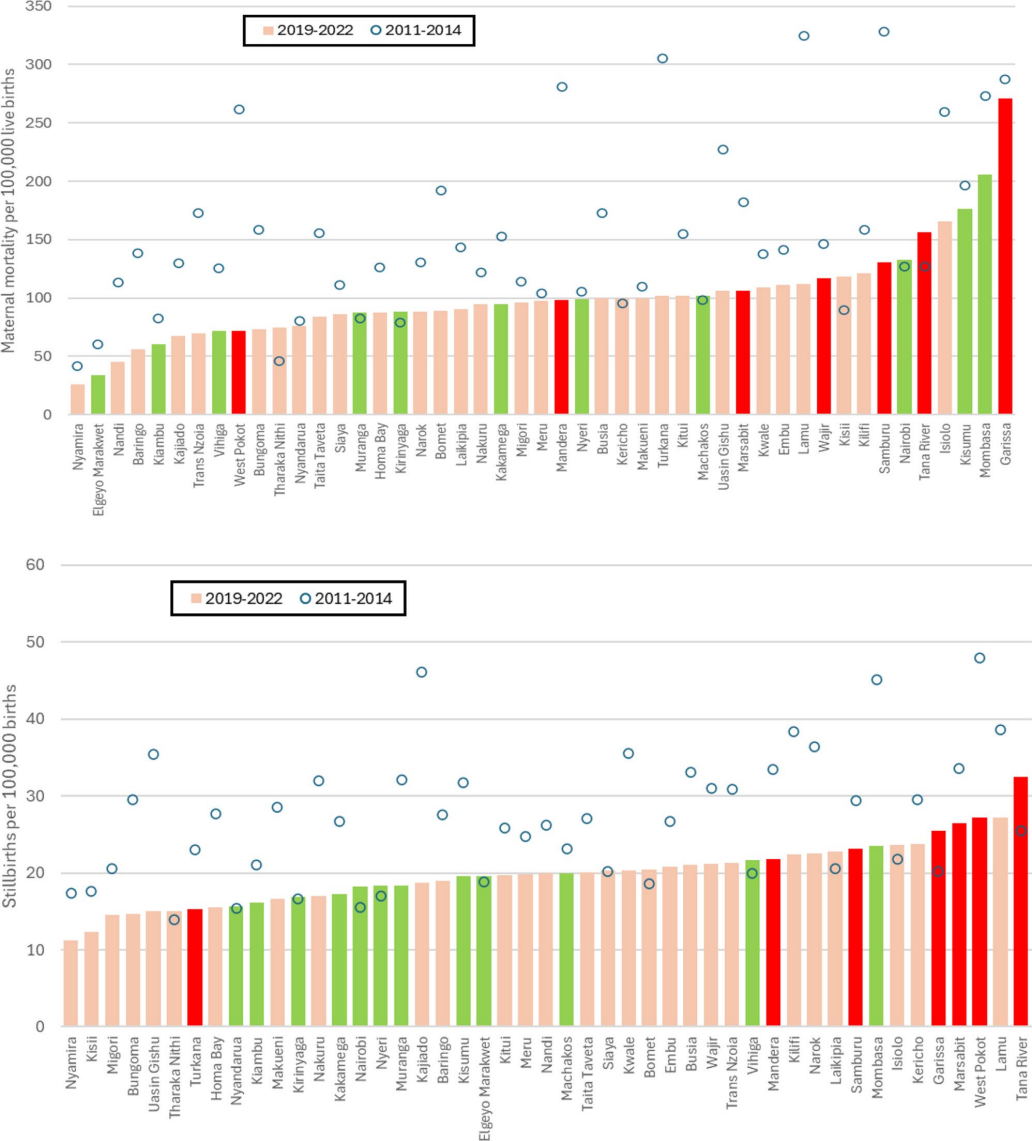
County levels and trends in institutional maternal mortality ratio (upper panel) and stillbirth rates (lower panel), 2011-14 and 2019-22 (counties lowest coverage group are red, highest coverage group green and intermediate coverage groups pink)

In 2019-22, only Garissa and Mombasa counties had iMMR above 200 (Fig. 3). Three counties (Kisumu, Isiolo and Tana River) were between 150 and 200, followed by Nairobi county with 132 per 100,000 live births. At the lower end, iMMR was below 50 in three counties (Nyamira, Elgeyo Marakwet and Nandi). Comparing 2019-22 with 2011-14, declines occurred in almost all counties. Eleven of the 47 counties had a decline of more than 50% between 2011- 14 and 2019-22, while an increase of more than 50% only occurred in Tharaka-Nithi.

There were also large differences in iSBR between counties, ranging from 11 in Nyamira to 32 per 1,000 births in Tana River. Five counties had more than 25 stillbirths per 1,000 births in health facilities for 2019-22 (Tana River, Lamu, West Pokot, Marsabit and Garissa), while four counties were below 15. Comparing 2019-22 with 2011-14, iSBR declined in 34 of the 47 counties (Fig. 3). Three counties experienced more than 50% declines in iSBR between the two periods (Uasin Gishu, Bungoma and Kajiado).

### Mortality by county coverage

For 2019–2022, the median iMMR was 111 and 92 per 100,000 live births in the groups with the lowest and highest delivery coverage, respectively (Table 1, panel B): a 19% difference. The national iMMR for 2019–2022 was 104 per 100,000 live births. The differences in iSBR were slightly larger: 24 per 1,000 births in the lowest coverage group, compared to 18 per 1,000 in the highest coverage group of counties (25% relative difference).

For 2019-22, iMMR and iSBR were positively associated (iMMR = 4.4*iSBR + 14.4, r^2^ = 0.18) (Appendix F). Institutional mortality levels were higher in the group of counties with the lowest coverage of live births by health facilities (Table 1, panel B). In bivariate regression analyses, the iMMR in counties was positively associated with the proportion of women with at least secondary education (*p* = 0.03), and weakly associated with institutional delivery coverage (*p* = 0.07) and poverty levels (*p* = 0.06) and not with fertility or health system characteristics (*p* > 0.10). The iSBR was significantly lower (*p* < 0.05) with all characteristics, including higher coverage of live births, higher educational levels, lower poverty levels, more health workers and more beds (Appendix G).

There were several counties with improbably high ratios of stillbirths to maternal death. Major outliers included Elgeyo Marakwet (ratio = 58), Nandi (44), Nyamira (44), West Pokot (38), Baringo (34), Trans Nzoia (31) and Vihiga (30). Another four counties had ratios between 25 and 30. These seven counties were all in the ten lowest iMMR counties. The lowest stillbirth to maternal deaths ratio was observed for Garissa (9), which is still within the plausibility range.

### Population mortality estimates

For Kenya, the estimated population MMR and SBR were close to the observed institutional rates as coverage of live births by health facilities was as high as 88%. For instance, if we assumed that the community MMR was two times higher than the iMMR for 2019–2022, the population MMR would be 117 per 100,000 live births, a small increase from the iMMR of 104 (Table 1, panel C). A similar scenario for stillbirths resulted in a population SBR of 22 per 1,000 births, 4 per 1,000 higher than the iSBR (Table 1, panel D).

The impact of different assumptions about the community to institutional mortality ratio is greatest in the low coverage counties, increasing the disparities between the four groups of counties. For instance, at a ratio of 3 the population MMR was 220 per 100,000 live births in the lowest coverage group and 97 in the highest coverage group. For stillbirths, the same ratio of 3 resulted in population rates of 46 and 19 per 1,000 births in the lowest and highest coverage groups, respectively. As expected, the impact of the adjustment for elevated community mortality on the population mortality was negligible for counties with coverage of at least 95% and small for those with coverage 85–94%.

### KHIS and MPDSR comparison

Until 2020, the number of maternal deaths in the MPDSR were much lower than in the monthly reporting system, partly because not all counties were reporting. In 2020, numbers were close or higher at the national level. In 2021 and 2022, the number of maternal deaths reported through the MPDSR were 10% and 14% higher than in the KHIS. If the highest annual number of maternal deaths reported by a county in either system is taken as the most complete estimate, the national iMMR would be 139, 124 and 126 per 100,000 live births in 2020, 2021 and 2022, respectively. In 2022, the number of deaths reported in the MPDSR was more than 50% higher than in the KHIS in 11 counties, while the reverse occurred for five counties. Details of the comparison can be found in Appendix E.

## Discussion

Our analysis of health facility data on maternal deaths and stillbirths in Kenya for 2011–2022 shows that it is possible to ascertain consistent trends in annual institutional maternal mortality and stillbirth statistics at national and county levels. In addition, we showed that, particularly given high coverage of births by health facilities, institutional mortality could become a critical input into the estimation of population levels of maternal mortality ratios and stillbirth rates especially if accompanied by efforts to estimate completeness.

The iMMR increase in 2020 may be associated with the temporary service disruptions that occurred in Kenya, as observed in many other countries [15]. In Kenya, rapid rebound of services may have prevented further mortality increases. The stagnation of the decline in recent years may partly be associated with better reporting of facility maternal deaths within the MPDSR but should also raise concerns about the quality of emergency obstetric care. Many women still deliver in smaller health facilities with limited intervention opportunities in case of complications, which mainly affects the poorest and rural women. The 2022 Kenya DHS shows that Caesarean section rates among women in the poorest 20% of households were 5.4%, which is often considered inadequate to meet the demand for this life-saving intervention [13]. 

Kenya and its 47 counties experienced a decline in iMMR and iSBR during the study period 2011–2022. By 2022, there were 99 maternal deaths per 100,000 live births and 17 stillbirths per 1,000 births in health facilities. Maternal mortality in health facilities was lower during 2019–2022 than 2011–2014 in 39 of the 47 counties. The national iMMR decline stagnated from 2018 at about 100. In 2020, there was a small increase to 117 which may have been associated with the COVID-19 pandemic. Stillbirth rates were lower in 2019–2022 compared to 2011–2014 in 35 counties. The decline continued in recent years from a peak of 28 per 1,000 births in 2012, with little impact of the pandemic in 2020–2021.

The plausibility of these results was assessed in multiple ways. The consistency of annual reports on live births, maternal deaths and stillbirths was good with only few extreme outliers to which we applied a correction. The consistency between two independent reporting systems for maternal deaths, the monthly aggregate KHIS and the continuous individual level MPDSR, was fairly good from 2020. In 2022, however, the individual reporting included 14% more maternal deaths. This difference was not considered due to the inclusion of community deaths in the MPDSR, which are supposed to be reported by the community health units to the health facility, as community death reporting is still very limited. It therefore may be indicative of underreporting in the KHIS. Better integration of the KHIS and MPDSR reporting systems is needed and an individual reporting system with additional variables on women’s characteristics and the cause of death, would be the best way forward.

Second, data quality assessment was done by considering the plausibility of the consistency between maternal death and stillbirth reporting. The national ratio of stillbirths to maternal death was 18 for 2019–2022 with little variation by county levels of institutional birth coverage, which was within our plausibility range. However, there were seven counties with improbably high ratios (30 or greater) and low MMR. These findings are suggestive of underreporting of maternal deaths in these counties. The ratio may vary by level of mortality but our review of relevant studies in eastern Africa did not provide evidence for such variation (Appendix B). Further work on plausible reference values for this ratio.

Third, county estimates of institutional mortality were higher evaluated by considering their levels of institutional birth coverage, fertility, poverty and health systems development. Less-developed counties, mostly located in the northern and northwestern Kenya, however, had only marginally higher institutional mortality than more developed counties in for instance the central part of Kenya. The main exception was Garissa which had an iMMR of 271 per 100,000 live births. Counties with near universal coverage of live births institutions had a wide range of iMMR for 2019–2022. This included the cities of Nairobi, Mombasa and Kisumu which were among the six highest iMMR counties, presumably due to a relatively higher share of complicated deliveries and higher number of referral facilities, as maternal deaths are reported by place of death and not place of residence. The association of institutional mortality with county socioeconomic and health system characteristics was generally weak for iMMR but much stronger for iSBR. Even though there is no simple association between institutional mortality and coverage and other county characteristics, these findings are suggestive of greater underreporting of maternal deaths than stillbirths in especially the less-developed counties.

To illustrate the impact of selective underreporting, we assumed that the low iMMR observed in seven of the eight less-developed counties in group 1, located in north and northeast Kenya, had the same iMMR as Garissa in that group (271). The national iMMR would increase by 13%, a relatively modest increase due to the smaller population sizes of these counties.

Lastly, we estimated population levels of maternal mortality and stillbirth rates from institutional mortality rates, coverage of live births by health facilities, and assumptions about the ratio of community to institutional mortality. Because institutional live birth coverage is high, the impact of different assumptions on elevated community mortality compared to institutional mortality is small. Research studies suggest that community to institutional mortality ratios are commonly in the range of 1.5-2.0, and rarely higher than 3 (Appendix C). Using a range of 1.5-3.0, national population maternal mortality would be 110–129 per 100,000 live births and stillbirth rate of 20–23 per 1,000 births.

These results are a long way from other population estimates of mortality for Kenya. The 2020 UN estimate for maternal mortality for Kenya was 530 (80% uncertainty interval: 328–750) which was two times higher than neighbouring Uganda and Tanzania [8]. The 2019 population census estimated 355 per 100,000 live births, based on recent deaths in the household, but major issues with the reporting of deaths and births were observed [15], and the reported county mortality levels were often counterintuitive. The observed levels are also incompatible with Global Burden of Disease study’s county estimates of maternal mortality which published highly improbable results with, for instance, one of the more developed counties, Nyeri, having the highest MMR (739) and a pastoralist-dominated county, West Pokot, the lowest (37) in 2016 [16]. 

With an iMMR of 104, as computed for 2019–2022, institutional birth coverage of 88% and a population estimate of MMR of 355, the community MMR would have to be 2196 per 100,000 live births, or 21 times higher than institutional mortality. This is highly unlikely. Our review of the limited number of published studies showed that community mortality was mostly 1.5-2.0 times higher than institutional mortality but no extreme ratios have been reported.

The iSBR was 18 per 1,000 births for 2019–2022, which was close to the population estimate of the UN for 2021 (18.5, 95% uncertainty interval 17.4, 19.9) [17]. The UN estimates however did not show a decline (2011 was 18.2 per 1,000 births (15.0, 21.9)) which differs from the steady decline of institutional SBR observed in the health facility data. At community to institutional mortality ratios of 1.5-2.0, the population SBR in Kenya ranges from 20 to 22 per 1,000 births for 2019–2022.

The main reasons for the large discrepancies between population MMR estimates based on only surveys and censuses and a population MMR derived from facility data are twofold. First, global estimates for countries tend to be based on limited survey and census data on maternal mortality with large uncertainty and long reference periods, with known limitations [1–4]. The 2020 UN estimate for Kenya is heavily driven by a statistical model, as no reliable national data were available after the 2014 DHS [8]. The Kenya MMR estimate for 2020 is widely believed to be too high, especially in comparison with estimates for neighbouring countries. Kenya’s population MMR for 2020 was more than two times higher than for Tanzania and Uganda, which seems unlikely given that most other relevant health indicators do not differ much among the three countries.

On the other hand, the population MMR primarily based on the iMMR from health facility data is likely to be too low, as the unadjusted iMMR input does not take full account of underreporting of deaths in the facility reporting system. Incomplete reporting of maternal deaths in health facilities is likely a major issue, as has been observed in even death registration systems in high-income settings, resulting in upward adjustments of the number of reported deaths [8, 18–20]. Underreporting of maternal deaths is common as facility deaths outside of the maternity wards are easily missed. This includes deaths in pregnancy (e.g. abortion, ectopic pregnancy, malaria) or postpartum after discharge (e.g., sepsis, eclampsia, indirect causes). The biases in institutional reporting of maternal deaths do not necessarily affect the iMMR trends as long as underreporting does not change over time but do influence the population estimates from iMMR.

Our study has several limitations. The trends in iMMR and iSBR have to be interpreted with caution as completeness of reporting may vary between years (and counties) and no year-by-year adjustments could be made. Our methods to assess data quality are novel but based on limited empirical data. Notably, the expected ranges for the stillbirth to maternal death ratio and the scenarios for the community to maternal mortality ratio are based on limited number of relevant empirical studies. Further work is needed to fill these gaps. In addition, we were not able triangulate our results with other facility-based mortality studies in Kenya or with recent population-based studies at national or county levels.

Levels of underreporting are likely to vary considerably between and within countries, as has been shown in low mortality countries [18]. Therefore, systematic assessment of the level of underreporting of institutional deaths from the maternity and other wards is needed. We used several analytical methods to assess the mortality reporting by counties. Our data quality assessment focused on analytical methods and falls short of a method to adjust for incomplete mortality reporting. This can only be done by developing a system to regularly assess the completeness of the data. This should include facility assessments (including record reviews and mortuary data to capture other wards) and cause-specific reviews of deaths to assess differential underreporting reporting at health facility and county levels.

In conclusion, our analysis provides a comprehensive assessment of 12 years of subnational maternal mortality and stillbirth rates in health facilities in Kenya. Institutional maternal mortality ratio and stillbirth rates have decreased considerably in all counties, but the maternal mortality decline has stagnated since 2018 at levels of mortality for both indicators that are indicative of critical deficits in the quality of care. Further methodological work is needed to assess facility data quality. We explored new approaches for the assessment of data quality and for triangulation of institutional and population mortality estimates. Efforts to improve the completeness and accuracy of death reporting by facilities is not only critical for monitoring the quality of care but can also become a critical input in the estimation of population levels of mortality, especially in populations with high delivery coverage, as in the case of Kenya.

## Electronic supplementary material

Below is the link to the electronic supplementary material.

Supplementary Material 1

## Data Availability

The datasets analyzed during the study are not publicly available (since the KHIS is not an open data source) but are available from the corresponding author on reasonable request.
