# Supplementary material for: Trends in maternal mortality and stillbirths by county in health facility data, Kenya, 2011-2022
Source: BMC Pregnancy Childbirth. 2025 Sep 11;25:932. doi: 10.1186/s12884-025-07726-6 (PMC12427102; doi:10.1186/s12884-025-07726-6)
Supplement: Supplementary file 1 — Supplementary Material 1 [file 12884_2025_7726_MOESM1_ESM.docx]

# **Trends in maternal mortality and stillbirths by county in health facility data, Kenya, 2011-2022**

Rose Muthee^1^, Martin Mutua^2^, Hannah Kagiri^1^, Helen Kiarie^1^, Simon Muchemi^1^, Scola Wabwire^1^, Ties Boerma^3^

1 Ministry of Health; 2 African Population and Health Research Center; 3 University of Manitoba

**Appendix**

**Appendix A: Assessment and adjustment reported births and deaths**

Time series analysis was used to calculate the relative difference between the year's reported number and an expected number based on the surrounding years. We computed the relative difference from the median value for the 2 years before and 2 years after the index year to flag questionable or problematic data: ((annual value – median) / median) * 100%. For 2011 and 2022 we used the two following / preceding years to compute the expected value. For 2012 and 2021 we used 2011-2013-2014-2015 and 2018-2019-2020,2022 respectively.

We used the relative difference to identify extreme outliers that deserved further inspection. The thresholds to identify major outliers differ for the three events as the numbers of reported events are very different. For live births we selected 25%, for maternal deaths 200% and stillbirths 100% relative difference from the expected value.

*Livebirths*

Figure A.1 shows the trends for live births. Nairobi is reported separately from the other counties because of its higher numbers. There was a major increase in the number of live births in the health facilities in the country over the period 2011- 2022 with most counties having at least 5,000 live births per year. The dip in 2017 is associated with a national health worker strike.

There were only seven district annual numbers of live births (1.2%) where the relative difference with the expected value exceeded 25%, and none above 30%. We did not make any corrections to the reported live birth data.

**Figure A.1 number of reported live births by year, Kenya, Nairobi and the remaining 46 counties, 2011-2022, KHIS**

******

*Maternal deaths*

During the period 2011- 2022, an average of 1094 maternal deaths were reported by health facilities through the routine system KHIS nationally. There was good consistency of the data for the two indicators over the period in most counties. 22 annual county values were more than 100% different from the median based on the surrounding years (3.9%). Six annual values differed at least 200% from the expected value. Based on an inspection of the full time series, including live births and stillbirths, only data for Siaya county in 2021 and 2022 were adjusted and given the median value of 2019-2020 for each year (26 deaths replaced 9 in 2021 and 100 reported deaths in 2022).

**Figure A.2: Number of maternal deaths by year, Kenya, Nairobi and the remaining 46 counties, reported in the KHIS 2011-2022**

For stillbirths there were 28 annual values that differed at least 100% from the expected value and eight county years with at least 200% difference. We inspected time series, and made corrections for eight counties, by imputing the median expected value for the year with the extreme value.

**Figure A.3: Number of stillbirths by year, Kenya, Nairobi and the remaining 46 counties, reported in the KHIS 2011-2022**

**Appendix B: Ratio stillbirths to maternal deaths in health facilities – expected values**

Maternal deaths, stillbirths and early neonatal death have several common causes, particularly during the perinatal period and related to maternal health. The quality of maternal care is a critical determinant of mortality risks for women, fetus and neonate. Therefore, we expect a positive association between the mortality rates.

At the population level, maternal mortality ratios are highly correlated with stillbirth and neonatal mortality rates.^[[1]](#footnote-1)^ The ratio of stillbirth and neonatal deaths combined to maternal deaths increased from less than 20, to over 75 as mortality declined in historical data from today’s high-income countries and in the UN estimates.^[[2]](#footnote-2)^ The country reporting of neonatal deaths before discharge is highly variable in most DHIS2 and needs a separate assessment. It is not included in this assessment.

We searched the literature for studies that would allow us to estimate a range of plausible values of the stillbirth to maternal death ratio. The main inclusion criteria for published studies include: (1) a systematic effort to maximize the completeness of the data on maternal deaths and stillbirths (2) We did not a priori exclude studies that provided an intervention but consider them on a case-by-case basis to assess the suitability for inclusion (3) at least 5000 livebirths (4) no tertiary hospital. We only included studies in sub-Saharan Africa.

The range of the ratio stillbirths to maternal deaths in health facilities was large, from about 5 to 30 stillbirths per maternal death:

- Dar es Salaam, 5 facilities, maternity registers plus data quality component, 2020, 36758 livebirths: MMR was 111 per 100,000 births, stillbirth 27.7 per 1,000 births; ratio 25.^[[3]](#footnote-3)^
- Dar es Salaam, 22 facilities, registers, interventions, over 70,000 livebirths each year, 2012, 2015 and 2019, MMR 150-80-77 and SBR 27-26-21, ratios 18, 33 and 27.^[[4]](#footnote-4)^
- Njombe district, Tanzania: 20 facilities in 2 districts, using DHIS2 data, 2016-2018, with interventions: combining the 3 years 8442 live births, 5 maternal deaths (MMR 59), and a ratio of 9.^[[5]](#footnote-5)^
- Upper East region in Ghana, 24 facilities, 3 districts, using DHIS2 data, 2016-2018, with interventions: combining the 3 years, 28,112 livebirths, MMR 263, SBR 23, ratio 10.^[[6]](#footnote-6)^
- Tanzania, 6 regions, 36 facilities, hospitals and health centres, 2021 baseline, 67690 deliveries, MMR 244, SBR 14.6, ratio 6.^[[7]](#footnote-7)^ The repeat assessment a year later showed a major decline in MMR (to 72) but not SBR, resulting in a ratio increase to 27.
- Kigoma Tanzania: all health facilities in one region, interventions, 2013 and 2018, 38,367 and 85045 live births, resp., MMR 303 and 174, SBR 27 and 13, ratios 9 and 7.^[[8]](#footnote-8)^
- Uganda, 128 facilities, routine reporting + data quality component, interventions, 2011-12, 2012-13 and 2016, over 30,000 LB, MMR from 534 to 354 and 203, SBR 31, 25 and 27. Ratio 6, 7 and 9.^[[9]](#footnote-9)^
- Zambia, 120 facilities, same years and approach as Uganda, interventions: 2011-12, 2012-13 and 2016, over 20,000 LB: MMR 310, 202, 231 and SBR 31, 25, 20. Ratios 10, 13 and 9.^[[10]](#footnote-10)^ ^[[11]](#footnote-11)^

There are major differences between the study sites, but less variability between years within the same studies. The major exception was the 6-district study in Tanzania where the ratio increased from 6 to 27 due to a plunging MMR but not SBR. Combining the results for all years (before and after intervention) from the main studies shows that 6 of the 7 studies have a ratio of stillbirth to maternal deaths in health facilities within the range of 7 to 11, with only Dar es Salaam as a major exception (ratio of 24).

**Figure B1. Ratio of stillbirths to maternal deaths, health facility studies, sub-Saharan Africa**


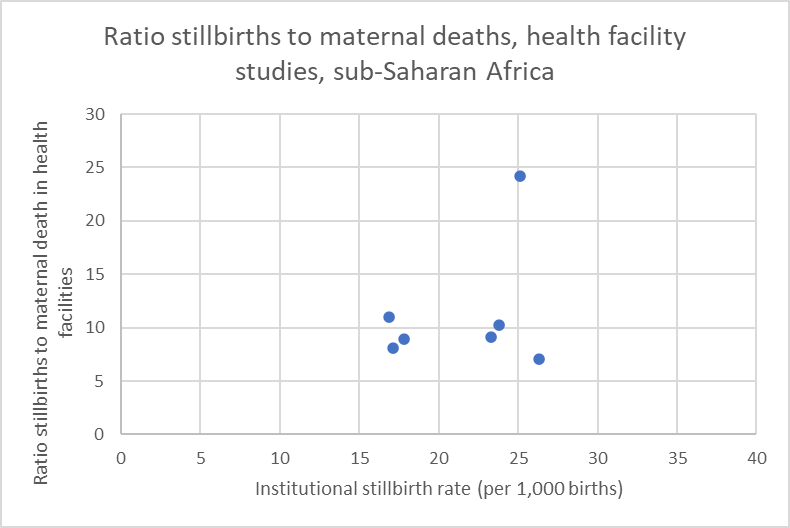


Table B.1: Mortality levels and ratios in selected studies

|  | MMR per 100,000 births | Stillbirths per 1000 births | Ratio stillbirths to maternal death |
| --- | --- | --- | --- |
| Dar es Salaam | 103 | 25.1 | 24.2 |
| Njombe, Tanzania | 200 | 17.8 | 8.9 |
| 6 districts, Tanzania | 153 | 16.9 | 11.0 |
| Kigoma, Tanzania | 210 | 17.1 | 8.1 |
| Upper East, Ghana | 257 | 23.3 | 9.1 |
| 3 districts, Uganda | 292 | 26.3 | 7.1 |
| 3 districts, Zambia | 226 | 23.8 | 10.2 |

Based on a range of historical and recent population-based and health facility studies, we considered a broad range of stillbirths to maternal deaths between 6-30 as a plausible range for the ratio.^^[[12]](#footnote-12)^^ A low ratio would be due underreporting of stillbirths but not maternal deaths, while a high ratio would most likely be major underreporting of maternal deaths. If maternal deaths are more likely to occur in hospital than stillbirths, given that problems are more “visible”, then the institutional ratios are expected to be lower than population ratios.

**Appendix C: Ratio of community to institutional maternal mortality ratio – expected values**

As the coverage of deliveries by health facilities increases, the institutional maternal mortality ratio (iMMR) becomes a critical input into the estimation of population levels of maternal mortality (pMMR). The pMMR is the sum of the iMMR and the community MMR (cMMR), weighted by the coverage of live births in institutions. Therefore, the computation of the pMMR from iMMR depends on the ratio cMMR / iMMR.

The cMMR is often unknown and difficult to estimate. Most surveys, including the DHS sibling survival history, do not include a question on place at death. Only a limited number of studies have data on place at death and can be used to estimate the ratio.

The Figure and Table below summarizes the results of studies in 6 countries in sub-Saharan Africa and 2 countries in South Asia that allowed the computation of the ratios.

Figure C.1: Ratio community to institutional maternal mortality ratio by institutional birth coverage


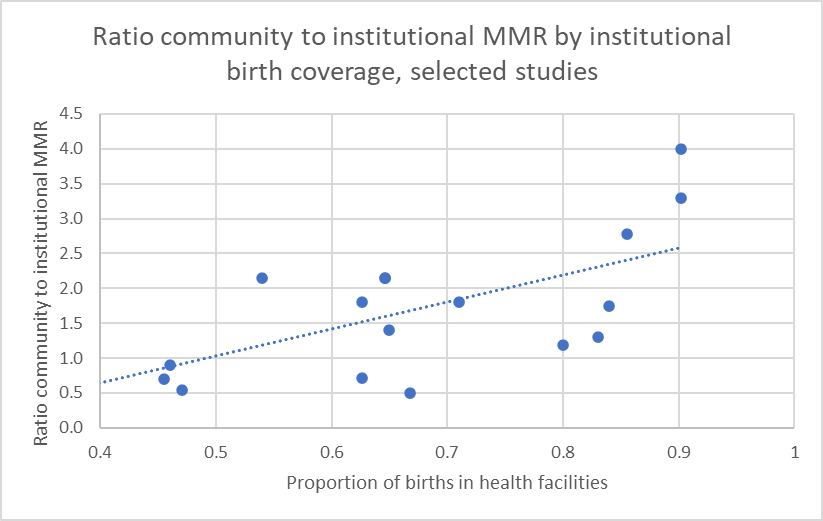

**Mozambique**

The sample vital statistics system (COMSA) found that 46% of maternal deaths occurred in health facilities in 2019-2020.^[[13]](#footnote-13)^ The DHS 2022/23 showed that 64.6% of live births were in health facilities. The pMMR in the DHS for the seven years preceding the survey was 233 per 100,000 live births.^[[14]](#footnote-14)^ The COMSA reported a much lower MMR for 2019-2020: 119.^[[15]](#footnote-15)^ The ratio cMMR to iMMR is 2.1.

The 2007 census included a follow-up survey with verbal autopsy. The PMMR was estimated at 500 per 100,000 live births. 37% of maternal deaths occurred in hospitals.^[[16]](#footnote-16)^ Based on the DHS 2003 and DHS 2011, we estimated 54% of live births occurred at health facilities. The ratio is then 2.1.

**Tanzania**

In Tanzania, three studies allow an assessment of the ratio. The first is a survey in southern Tanzania (Lindi-Mtwara) in 2013.^[[17]](#footnote-17)^ The pMMR was estimated at 500. Out of 37 maternal deaths reported in the survey for mid-2011 to mid-2013, 77% occurred in health facilities. According to the TDHS 2016, the institutional live birth rate was 81% in Lindi and Mtwara for the three-year period before the survey. The cMMR/iMMR ratio was 1.2.

The second study is the Magu Health and Demographic Surveillance system in northwest Tanzania. The MMR for 2015-2022 was 280 per 100,000 live births. According to the verbal autopsies, 75% of the 24 maternal deaths during 2015-2022 occurred in health facilities. The institutional live birth rate was 88% for 2019-2020, higher than the regional average of 80% in TDHS 2022 and 53% in TDHS in TDHS 2016. Here, we use 84% coverage. The ratio was 1.8.

The Sumve survey in northwest Tanzania was conducted by DHS to improve the survey instruments.^[[18]](#footnote-18)^ In this rural areas the pMMR was estimated at 463 per 100,000 live births, with 38% of live births in health facilities and 36% of maternal deaths in health facilities. The CMMR/iMMR ratio was 1.1.

**Uganda**

This was part of an intervention study with baseline and endline data collection. In 4 districts, a RAMOS study was conducted in 2011-2012 and again in 2016 (Jan-Dec) to measure population MMR, as well as health facility assessments (105 facilities). The RAMOS in 2011 used community registers to identify deaths for the preceding 18 months, followed up with a verbal autopsy interview. In 2016, household interviews were conducted to capture all relevant deaths since Jan 2016. Live births were estimated from other sources.

The institutional delivery rate increased from 45.5% to 66.8%, while the iMMR declined from 534 to 300. The pMMR was 452 at baseline and 255 at endline. The cMMR can be computed from these data: 384 and 164, respectively. This results in ratios of 0.7 and 0.5.

For 2011-12 the place of death is also provided. 48% of maternal deaths were in health facilities which with a coverage of live births of 45.5% corresponds with a cMMR/iMMR ratio of 0.9.

**Zambia**

This was part of an intervention study with baseline and endline data collection. In 4 districts, community-level maternal mortality data were collected using household population censuses conducted in 2012 and 2017, using deaths in the last 12 months. The suspected underreporting of deaths was adjusted using demographic methods. Live births were estimated from other sources.

The institutional delivery rate increased from 63% to 90%, while the iMMR declined from 370 to 231. The pMMR was 480 in baseline and 284 at endline. The cMMR was high as can be computed from these data: 664 and 772, respectively. This results in ratios of 1.8 and 3.3.

Using the data on place of death, 70% died in health facilities in both rounds, but the percent of births in health facilities increased from 63% to 90%. This means that the ratio was 0.9 in 2010-11 and 4.0 in 2016.

**Zimbabwe**

A RAMOS study was conducted in 11 districts in 2007-08 and 2018-19.^[[19]](#footnote-19)^ The second RAMOS was affected by major underreporting of community deaths and substantial adjustment was made to increase the number of maternal deaths, using the proportions of community to institutional maternal deaths from the first RAMOS to make the adjustments.^[[20]](#footnote-20)^

The institutional birth rate increased from 65% to 86%, the percent maternal deaths in health facilities was 68% in the first RAMOS. The MMR in the first RAMOS was 649, the ratio cMMR/iMMR 0.9. In the unadjusted data in the second RAMOS, 81% of maternal deaths occurred in health facilities which would correspond with a ratio of 1.4. Using the same proportion of maternal deaths in health facilities as in the first RAMOS (68%), the ratio is 2.8.

**Sierra Leone**

A sample registration system was run from 2018-2020.^[[21]](#footnote-21)^ The MMR was 510, and 75% of maternal deaths were reported to be in health facilities (maybe less because on the way may have been included). The DHS 2019 shows that 83% were institutional live births. The ratio is 1.3.

**Other**

National surveys in Bangladesh and Pakistan used deaths in the household in the last 3 years to estimate MMR.

In Bangladesh 2016 62% of maternal deaths occurred in health facilities, 19% at home and 19% on the way. The coverage of facility births was 47%, and MMR 196. The ratio is 0.5.

In Pakistan 2019, 58% of maternal deaths took place in health facilities, 18% at home, 22% in transit and 2.5% other. Institutional birth coverage was 71%, resulting in a ratio of 1.8.

**Appendix D: Associations between institutional live births coverage and county characteristics**

**Coverage of live births in health facilities has increased in all counties (Figure) and is strongly associated with all county characteristics (Table).**

**Figure D.1 Institutional coverage of live births (%) in the two years preceding the survey, counties, DHS 2014 and DHS 2022, Kenya**


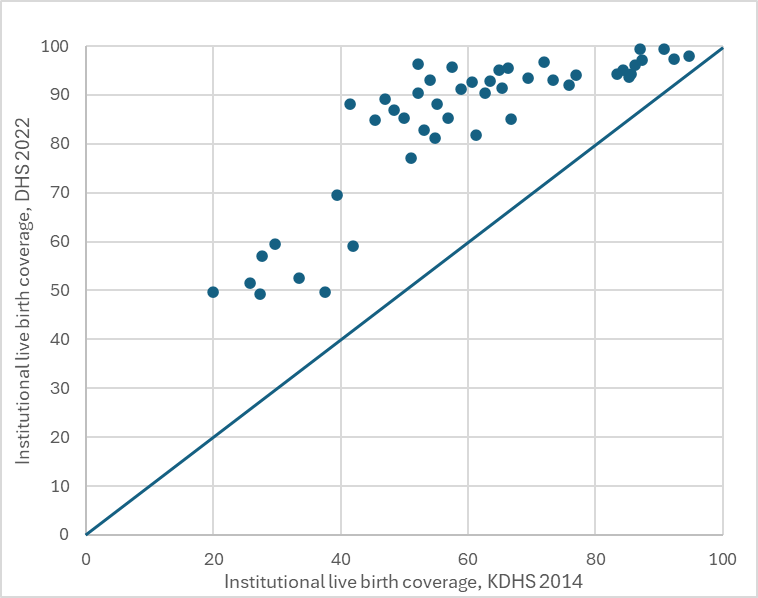


**Figure D.2 Institutional live birth coverage (DHS 2022) by district poverty headcount (KNBS, 2021) by county**

Coverage of live births is strongly associated with the poverty headcount ratio by county. Narok is a major outlier with a delivery coverage (69.6%) that is much lower than expected on the basis its poverty headcount (22%). It is possible that the poverty in Narok is underestimated.

The correlation matrix shows the strong association of all indicators with the coverage of live births in counties. Less poverty, more female education, lower fertility, are strongly associated with coverage, while health workers density and hospital beds density are moderately strongly associated with coverage.

**Table D.1. Correlation matrix of coverage of live births with county characteristics**

|  | delcov | poverty | educfem | tfrdhs | hrh2022 | beds2022 |
| --- | --- | --- | --- | --- | --- | --- |
| Coverage of live births | 1 |  |  |  |  |  |
| Poverty level | -0.8226 | 1 |  |  |  |  |
| Female education (secondary or higher) | 0.8347 | -0.7819 | 1 |  |  |  |
| Total fertility rate (DHS 2022) | -0.9019 | 0.7761 | -0.8489 | 1 |  |  |
| Health workforce density (doctors, nurses, midwives) | 0.4812 | -0.5224 | 0.5435 | -0.4832 | 1 |  |
| Hospital beds density | 0.4118 | -0.457 | 0.5221 | -0.4265 | 0.8809 | 1 |

**Appendix E Comparison KHIS and MPDSR**

From 2017 to 2019, the M(P)DSR reported lower numbers than KHIS but since 2020, about 10% more deaths are reported through MDSR than KHIS (Figure 4 and Table 1). The number of counties reporting in the MPDSR increased over time, and so did the numbers of maternal deaths. By 2022, the MPDSR numbers were at least 25% higher than the KHIS annual numbers in 20 of the 47 counties. Since underreporting is the main challenge, we selected the highest value of either system as the best reported number. As expected, this resulted in an increase of the total number of reported deaths in Kenya as a whole. The greatest increase was in 2022 (28%), followed by 2021 (24% higher than in KHIS). We also created a new variable ‘mdbest’ was created which is the maximum annual value for either the KHIS or the MPDSR maternal death estimates.

**Figure E.12: Comparison of number of maternal deaths reported in the KHIS and notified in the MPDSR Kenya, 2017-2022**


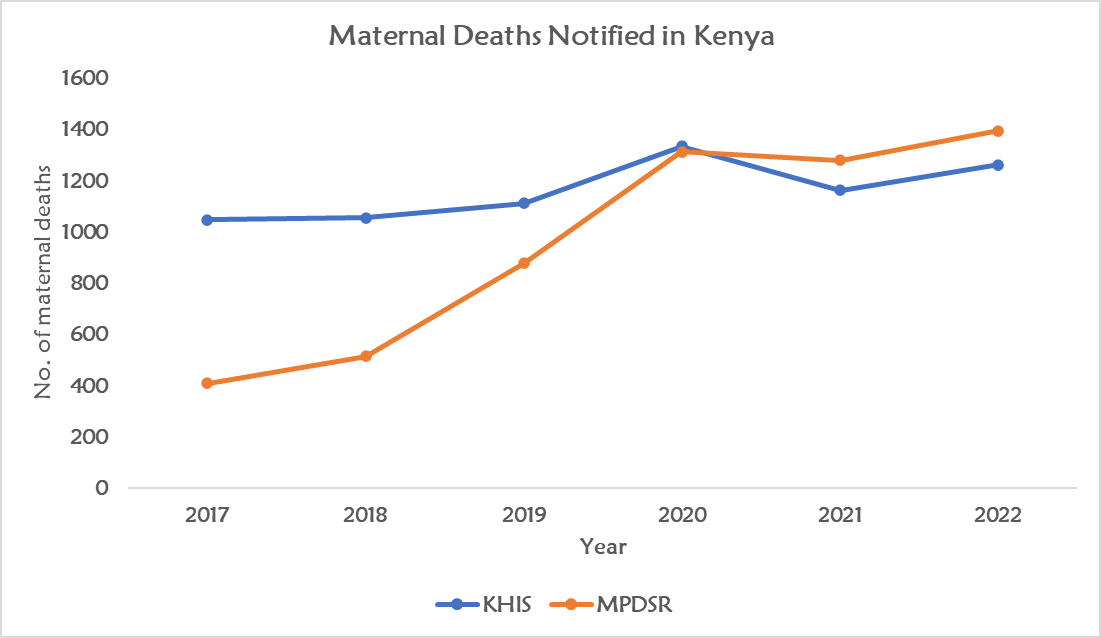


**Table E.1. Comparison of reported maternal deaths in KHIS and MPDSR, Kenya, 2017-2022**

|  | **2017** | **2018** | **2019** | **2020** | **2021** | **2022** |
| --- | --- | --- | --- | --- | --- | --- |
| Total KHIS, reported maternal deaths | 1048 | 1056 | 1113 | 1334 | 1179 | 1188 |
| Total MPDSR, reported deaths | 410 | 514 | 877 | 1313 | 1280 | 1395 |
| Combined, based on highest value by county | 1090 | 1101 | 1256 | 1586 | 1456 | 1520 |
| Increase compared to KHIS (%) | 4.0 | 4.3 | 12.8 | 18.9 | 23.5 | 27.9 |
|  |  |  |  |  |  |  |
| Counties (N) | 47 | 47 | 47 | 47 | 47 | 47 |
| No MPDSR data (N) | 17 | 12 | 6 | 4 | 5 | 3 |
| MPDSR >=25% or higher than KHIS (N) | 1 | 1 | 3 | 12 | 18 | 20 |
| Within range of plus or minus 25% (N) | 8 | 10 | 15 | 19 | 16 | 14 |
| MPDSR <25% or lower (N) | 21 | 24 | 23 | 12 | 8 | 10 |

**Appendix F: Association between MMR and SBR in health facilities, 2019-2022**

Maternal mortality was positively associated with stillbirth mortality but with considerable variation. Counties in green are part of group 4 (delivery coverage >= 95%) and in orange are part of group 1 (<60% coverage).

Figure F.1 Maternal mortality ratio per 100,000 live births by stillbirth rate per 1,000 births, by county, KHIS data


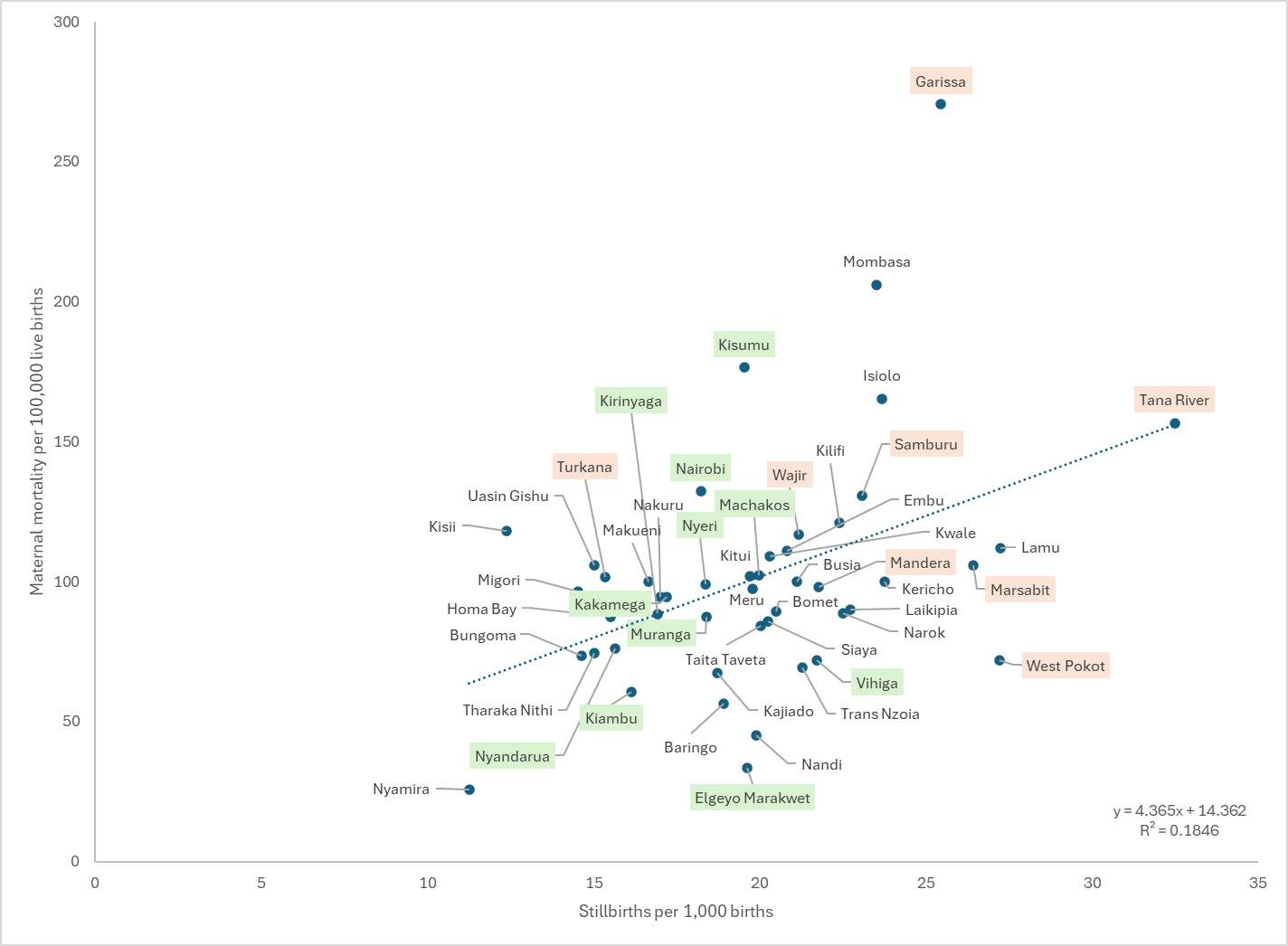


**Table F.2: County maternal mortality ratio per 100,000 live birth and stillbirths per 1,000 births, 2011-2014, 2015-18, 2019-22 with relative change between the first and last periods, KHIS data.**

|  | MMR 2011-14 | MMR 2015-18 | MMR 2019-22 | %change |  | SBR 2011-14 | SBR 2015-18 | SBR 2019-22 | %change |
| --- | --- | --- | --- | --- | --- | --- | --- | --- | --- |
| Baringo | 138 | 82 | 56 | 59 |  | 28 | 20 | 19 | 31 |
| Bomet | 192 | 112 | 89 | 53 |  | 19 | 26 | 20 | -10 |
| Bungoma | 158 | 82 | 74 | 54 |  | 30 | 16 | 14.63 | 50 |
| Busia | 173 | 102 | 100 | 42 |  | 33 | 28 | 21 | 36 |
| Elgeyo Marakwet | 61 | 54 | 34 | 44 |  | 19 | 20 | 20 | -4 |
| Embu | 141 | 100 | 111 | 21 |  | 27 | 28 | 21 | 22 |
| Garissa | 287 | 289 | 271 | 6 |  | 20 | 24 | 25 | -26 |
| Homa Bay | 126 | 91 | 87 | 31 |  | 28 | 18 | 16 | 44 |
| Isiolo | 259 | 152 | 165 | 36 |  | 22 | 25 | 24 | -9 |
| Kajiado | 130 | 97 | 68 | 48 |  | 46 | 23 | 19 | 59 |
| Kakamega | 153 | 98 | 95 | 38 |  | 27 | 20 | 17 | 36 |
| Kericho | 96 | 83 | 100 | -5 |  | 30 | 27 | 24 | 20 |
| Kiambu | 82 | 70 | 61 | 26 |  | 21 | 17 | 16 | 23 |
| Kilifi | 159 | 100 | 121 | 24 |  | 38 | 29 | 22 | 42 |
| Kirinyaga | 79 | 82 | 89 | -12 |  | 17 | 16 | 17 | -2 |
| Kisii | 90 | 95 | 118 | -31 |  | 18 | 19 | 12.39 | 30 |
| Kisumu | 197 | 184 | 177 | 10 |  | 32 | 24 | 20 | 38 |
| Kitui | 155 | 105 | 102 | 34 |  | 26 | 27 | 20 | 24 |
| Kwale | 138 | 143 | 109 | 21 |  | 36 | 24 | 20 | 43 |
| Laikipia | 143 | 99 | 90 | 37 |  | 21 | 22 | 23 | -10 |
| Lamu | 325 | 253 | 112 | 65 |  | 39 | 34 | 27 | 29 |
| Machakos | 99 | 66 | 102 | -4 |  | 23 | 17 | 20 | 14 |
| Makueni | 110 | 93 | 100 | 9 |  | 29 | 23 | 17 | 42 |
| Mandera | 281 | 90 | 98 | 65 |  | 33 | 24 | 22 | 35 |
| Marsabit | 182 | 166 | 106 | 42 |  | 34 | 27 | 26 | 21 |
| Meru | 104 | 155 | 98 | 6 |  | 25 | 22 | 20 | 20 |
| Migori | 114 | 81 | 96 | 16 |  | 21 | 15 | 15 | 29 |
| Mombasa | 273 | 177 | 206 | 24 |  | 45 | 34 | 24 | 48 |
| Muranga | 83 | 74 | 87 | -6 |  | 32 | 17 | 18 | 43 |
| Nairobi | 127 | 147 | 132 | -4 |  | 16 | 21 | 18 | -18 |
| Nakuru | 122 | 92 | 94 | 23 |  | 32 | 19 | 17 | 47 |
| Nandi | 114 | 64 | 45 | 60 |  | 26 | 25 | 20 | 24 |
| Narok | 131 | 106 | 89 | 32 |  | 36 | 31 | 23 | 38 |
| Nyamira | 41 | 38 | 26 | 38 |  | 17 | 10 | 11 | 35 |
| Nyandarua | 80 | 68 | 76 | 5 |  | 15 | 14 | 16 | -1 |
| Nyeri | 106 | 111 | 99 | 6 |  | 17 | 19 | 18 | -8 |
| Samburu | 328 | 146 | 131 | 60 |  | 29 | 19 | 23 | 22 |
| Siaya | 111 | 92 | 86 | 23 |  | 20 | 21 | 20 | -1 |
| Taita Taveta | 155 | 116 | 84 | 46 |  | 27 | 24 | 20 | 26 |
| Tana River | 127 | 250 | 157 | -24 |  | 25 | 36 | 32 | -28 |
| Tharaka Nithi | 46 | 97 | 74 | -61 |  | 14 | 20 | 15 | -8 |
| Trans Nzoia | 173 | 137 | 69 | 60 |  | 31 | 23 | 21 | 31 |
| Turkana | 305 | 227 | 102 | 67 |  | 23 | 21 | 15 | 33 |
| Uasin Gishu | 227 | 116 | 106 | 53 |  | 35 | 24 | 15 | 58 |
| Vihiga | 125 | 181 | 72 | 43 |  | 20 | 19 | 22 | -9 |
| Wajir | 146 | 116 | 117 | 20 |  | 31 | 20 | 21 | 32 |
| West Pokot | 262 | 119 | 72 | 72 |  | 48 | 34 | 27 | 43 |

1. McClure EM, Goldenberg RL, Bann CM. Maternal mortality, stillbirth and measures of obstetric care in developing and developed countries Int J Gynaecol Obstet. 2007;96:139-46. [↑](#footnote-ref-1)
2. Boerma T, Campbell OMR, Amouzou A, Blumenberg C, Blencowe H, Moran A, Lawn JE, Ikilezi G. Maternal mortality, stillbirths, and neonatal mortality: a transition model based on analyses of 151 countries. Lancet Glob Health. 2023 Jul;11(7):e1024-e1031. [↑](#footnote-ref-2)
3. Sequeira Dmello B, John TW, et al. Incidence and determinants of perinatal mortality in five urban hospitals in Dar es Salaam, Tanzania: a cohort study with an embedded case-control analysis. BMC Pregnancy Childbirth. 2024 Jan 13;24(1):62.  [↑](#footnote-ref-3)
4. Sequeira Dmello B, Sellah Z, et al. Learning from changes concurrent with implementing a complex and dynamic intervention to improve urban maternal and perinatal health in Dar es Salaam, Tanzania, 2011-2019. BMJ Glob Health. 2021 Jan;6(1):e004022. [↑](#footnote-ref-4)
5. Manu A, Billah SM, Williams J et al. Institutionalising maternal and newborn quality-of-care standards in Bangladesh, Ghana and Tanzania: a quasi-experimental study. BMJ Glob Health. 2022 Sep;7(9):e009471. [↑](#footnote-ref-5)
6. Manu et al. 2022 [↑](#footnote-ref-6)
7. Ersdal H, Mdoe P, Mduma E, et al. "Safer Births Bundle of Care" Implementation and Perinatal Impact at 30 Hospitals in Tanzania-Halfway Evaluation. Children (Basel). 2023 Jan 30;10(2):255.  [↑](#footnote-ref-7)
8. Dominico S, Serbanescu F, Mwakatundu N, et al. A Comprehensive Approach to Improving Emergency Obstetric and Newborn Care in Kigoma, Tanzania. Glob Health Sci Pract. 2022 Apr 29;10(2):e2100485.  [↑](#footnote-ref-8)
9. Serbanescu F, Clark TA, Goodwin MM, et al. Impact of the Saving Mothers, Giving Life Approach on Decreasing Maternal and Perinatal Deaths in Uganda and Zambia. Glob Health Sci Pract. 2019 Mar 13;7(Suppl 1):S27-S47. [↑](#footnote-ref-9)
10. Central Statistical Office (CSO) [Zambia], University of Zambia Department of Population Studies (UNZA), and ICF. 2017. *2017 Zambia Maternal Mortality Endline Census in Selected Districts*. Rockville, Maryland, USA. [↑](#footnote-ref-10)
11. Serbanescu F, Clark TA, Goodwin MM, et al. Impact of the Saving Mothers, Giving Life Approach on Decreasing Maternal and Perinatal Deaths in Uganda and Zambia. Glob Health Sci Pract. 2019 Mar 13;7(Suppl 1):S27-S47. [↑](#footnote-ref-11)
12. Boerma T, Campbell OMR, Amouzou A, et al. Maternal mortality, stillbirths, and neonatal mortality: a transition model based on analyses of 151 countries. Lancet Glob Health. 2023 Jul;11(7):e1024-e1031. [↑](#footnote-ref-12)
13. Preliminary data from Agbessi Amouzou, Johns Hopkins University [↑](#footnote-ref-13)
14. Instituto Nacional de Estatística (INE) e ICF. 2024. *Inquérito Demográfico e de Saúde em Moçambique 2022–23*. Maputo, Moçambique e Rockville, Maryland, EUA: INE e ICF. [↑](#footnote-ref-14)
15. UN MMEIG. Maternal mortality Mozambique 2000-2020. Internationally comparable MMR estimates by the Maternal Mortality Inter-Agency Group (MMEIG): WHO, UNICEF, UNFPA, World Bank Group and the United Nations Population Division. [↑](#footnote-ref-15)
16. Mozambique National Institute of Statistics, U.S. Census Bureau, MEASURE Evaluation, U.S. Centers for Disease Control and Prevention. 2012. *Mortality in Mozambique: Results from a 2007–2008 Post-Census Mortality Survey*. Chapel Hill, USA: MEASURE Evaluation. [↑](#footnote-ref-16)
17. Data from Claudia Hanson, Karolinska Institute, Sweden [↑](#footnote-ref-17)
18. Bicego, George, Curtis Sian, Raggers Hendrik, Kapiga Saidi, Ngallaba S. Sumve Survey on Adult and Childhood Mortality, Tanzania, 1995: In-Depth Study on Estimating Adult and Childhood Mortality in Settings of High Adult Mortality. Calverton, Maryland: Macro International Inc. [↑](#footnote-ref-18)
19. Musarandega R, Cresswell J, Magwali T, et al. Maternal mortality decline in Zimbabwe, 2007/2008 to 2018/2019: findings from mortality surveys using civil registration, vital statistics and health system data. BMJ Glob Health. 2022 Aug;7(8):e009465.  [↑](#footnote-ref-19)
20. We corrected a computational error in the publication and used a population MMR of 193 for the second RAMOS. [↑](#footnote-ref-20)
21. Carshon-Marsh R, Aimone A, Ansumana R, et al. Child, maternal, and adult mortality in Sierra Leone: nationally representative mortality survey 2018-20. Lancet Glob Health. 2022 Jan;10(1):e114-e123.  [↑](#footnote-ref-21)
